# Treatment of Locally Advanced Gastric Cancer (LAGC): Back to Lauren’s Classification in Pan–Cancer Analysis Era?

**DOI:** 10.3390/cancers12071749

**Published:** 2020-07-01

**Authors:** Ina Valeria Zurlo, Michele Basso, Antonia Strippoli, Maria Alessandra Calegari, Armando Orlandi, Alessandra Cassano, Mariantonietta Di Salvatore, Giovanna Garufi, Emilio Bria, Giampaolo Tortora, Carlo Barone, Carmelo Pozzo

**Affiliations:** 1 Comprehensive Cancer Center, Università Cattolica del Sacro Cuore-IRCCS, 00168 Rome, Italy; mariaalessandra.calegari@policlinicogemelli.it (M.A.C.); alessandra.cassano@unicatt.it (A.C.); giovanna.garufi@unicatt.it (G.G.); emilio.bria@unicatt.it (E.B.); giampaolo.tortora@unicatt.it (G.T.); carlo.barone@unicatt.it (C.B.); 2 Comprehensive Cancer Center, Policlinico Universitario “Agostino Gemelli”-IRCSS, 00168 Roma, Italy; michele.basso@policlinicogemelli.it (M.B.); antonia.strippoli@policlinicogemelli.it (A.S.); armando.orlandi@policlinicogemelli.it (A.O.); disalvatore.mariantonietta@gmail.com (M.D.S.); carmelo.pozzo@policlinicogemelli.it (C.P.)

**Keywords:** gastric cancer, diffuse histology, intestinal histology, neo-adjuvant therapy, perioperative therapy, adjuvant therapy

## Abstract

*Background*: Guidelines recommend a perioperative approach in patients with stage II/III gastric cancer, but in real-life many patients receive immediate surgery followed by adjuvant chemotherapy (aCT). Although histologic subtypes may have different response to CT, no study has explored the influence of histotype on the efficacy of perioperative CT (pCT) or aCT. *Materials and methods*: The objective of the study was to evaluate the impact of clinicopathological features and histology (intestinal or diffuse) on survival according to strategy (pCT vs. aCT). The primary endpoint was overall survival (OS) and the secondary endpoint was event-free survival (EFS). *Results:* Out of 203 patients affected by LAGC, 83 received pCT and 120 aCT. At multivariate, histology and LVI in pCT cohort and positive resection margin in the aCT influenced both OS and EFS. No difference in EFS and OS was observed in relation to strategy. However, in the intestinal-type of pCT cohort survival outcomes were significantly higher compared to the aCT cohort, whereas in the diffuse-type were significantly worse in patients receiving pCT compared to those receiving aCT. *Conclusions:* Although retrospective and small-sized, this study suggests that the benefit of pCT might be limited to the intestinal-type. This hypothesis needs to be confirmed in prospective series.

## 1. Introduction

Although the incidence of gastric cancer (GC) has been substantially declining for several decades, it remains a major cause of cancer mortality due to poor prognosis. Until 2006, surgery was the only really effective strategy for patients with resectable locally advanced GC (LAGC), with an adequate D2 lymphadenectomy qualifying as the gold standard approach [1,2]. However, despite R0 resection, the rate of postoperative recurrence is high. Consequently, many efforts have been made to improve survival through administration of pCT or aCT [3,4,5,6,7,8]. The MAGIC and FFCD-FNCLCC 9703 trials showed the superiority of a perioperative strategy compared to surgery alone, due to tumor downstaging allowing better surgery, about 13% decrease of recurrence and improvement of survival [9,10]. On the other hand, many randomized clinical trials have compared surgery alone with aCT or adjuvant chemoradiotherapy (aCT-RT), but clear evidence of benefit is lacking because survival improvement was only modest and sometimes controversial [3,4,5,6,7,8,11,12,13]. The largest benefit from aCT and aCT-RT has been demonstrated in patients who underwent D0 or D1 surgery, while the same benefit is less clear in patients who underwent D2 lymphadenectomy [4,11,13,14]. More recently, combination regimens with docetaxel, oxaliplatin and fluorouracil (FLOT schedule) have demonstrated better outcomes in terms of DFS and OS, when compared to ECF/ECX in the perioperative setting [15]. 

Therefore, international guidelines recommend an adequate D2 lymphadenectomy eventually preceded by a perioperative strategy for all patients with stage II or III disease. However, despite evidence and recommendations, in real-life, many patients receive immediate surgery followed by aCT. 

GC is a heterogeneous entity and histology is one of the earliest recognized criteria used for subtyping GC and it is frequently considered as a prognostic factor [16]. According to Lauren’s classification, GC is divided into two histological entities characterized by different epidemiology, pathogenesis, biological features and clinical behavior: the intestinal and diffuse subtypes [17]. Intestinal-type tumors form gland-like structures; are strongly associated with severe atrophic gastritis, intestinal metaplasia and *Helicobacter Pylori* infection; and display a better survival [18,19,20]. On the other hand, diffuse histology is associated with cellular discohesion (due to the lack of cadherin E expression) and poor differentiation and is characterized by chemoresistance, rapid progression and poor prognosis. There are many conflicting retrospective data concerning the association between histology and treatment outcome in GC, therefore the Lauren’s classification cannot be acknowledged as predictive of response to currently used drugs [20,21].

In the modern era of precision medicine, other molecular classifications have gained prominence, in particular the comprehensive molecular characterization [22]. However, neither randomized nor prospective trials have been conducted selecting or stratifying patients according to molecularly defined categories. Similarly, no analysis has been carried out in order to evaluate whether histologic subtypes of LAGC may be differently affected by the strategy of treatment. 

Since it is widely accepted that different subtypes of GC represent conditions with different biologic and clinical characteristics, it is conceivable that they might respond in a not uniform way to different strategies of treatment. Thus, we have hypothesized that histology might predict a different benefit from pCT or aCT, allowing to define the optimal approach. The primary endpoint of this retrospective analysis was to compare overall survival of patients receiving perioperative chemotherapy according to histology and overall survival of patients receiving immediate surgery and adjuvant chemotherapy according to histology. 

## 2. Patients and Methods

### 2.1. Study Population

This is a retrospective monocentric study. Clinical records of all patients affected by LAGC treated with aCT or pCT at Medical Oncology Unit of the Fondazione Policlinico Universitario “A. Gemelli”-IRCCS, Rome, Italy, between January 2009 and January 2018 were reviewed. Inclusion criteria were: (1) histologically confirmed adenocarcinoma of the stomach; (2) gastro-esophageal junction, Siewert type 2 or 3 (GEJ) or non-cardia GC (stomach); (3) known histotype according to Lauren’s classification (intestinal or diffuse); (4) total or sub-total gastrectomy; (5) stage II or III (clinical staging for pCT and pathological staging for aCT); (6) CT administration in perioperative or adjuvant setting; (7) age ≥ 18 years; (8) no serious concomitant illnesses that could have affected treatment duration, short-time survival or the possibility of surgery; (9) performance status (PS) according to the Eastern Cooperative Oncology Group of 0 or 1; (10) adequate organ function (bone marrow, liver and kidney); (11) left ventricular ejection fraction (LVEF) of ≥50% for anthracycline-containing CT; and (12) informed consent to surgery and chemotherapy according to local practice. Patients whose histotype according to Lauren’s classification was not known or mixed-type, with gastro-esophageal junction, Siewert type 1, with medical history of metastatic disease or other cancers (with the exception of non-melanoma skin cancers and in situ cervical cancer) diagnosed within the previous 5 years, or who received upfront surgery without aCT due to clinical conditions or concomitant illness, were excluded. Patients were divided into two groups according to treatment strategy (perioperative and adjuvant) and outcome was evaluated according to histology (intestinal vs. diffuse).

All patient data were collected anonymously; the study was conducted in accordance with the Declaration of Helsinki and consent for chemotherapy was obtained by all patients, also including the consent for retrospective analysis of all clinical data, according to the Ethical Committee of the Catholic University School of Medicine. 

In the pCT group, T staging was determined using endoscopic ultrasonography (EUS) and N and M status were classified using a contrast-enhanced computer tomography (CE-CT) scan of the abdomen and chest. In the aCT group, TNM staging was assigned based on pathological examination and distant metastases were excluded by CE-CT.

### 2.2. Treatment Procedures

Both in pCT and aCT cohort the most frequently used regimens were triplet CT [epirubicin plus oxaliplatin plus capecitabine (EOX), or epirubicin plus cisplatin plus infusional 5-fluorouracil (ECF)] or platinum-containing doublet CT [leucovorin plus oxaliplatin plus bolus and infusional 5-fluorouracil (FOLFOX-6), capecitabine plus oxaliplatin (XELOX) or cisplatin plus 5-fluorouracil (CF)]. Only in few cases of aCT cohort, a fluoropyrimidine monotherapy was employed (DeGramont regimen). In the pCT cohort, restaging was accomplished using CE-CT scan before surgery. All patients underwent gastrectomy (total or subtotal according to tumor extension and location) with an adequate D2 lymphadenectomy. Margin resection was defined as R0 when no tumor was identified on microscopic examination of proximal, distal or circumferential margin and as R1 when microscopic margin involvement was demonstrated. In the pCT cohort, surgery was carried out within 6–8 weeks from the last CT course, whereas in the aCT cohort the first cycle of CT was administered within 8 weeks after surgery. Postoperative morbidity and mortality were recorded. All patients underwent follow-up according international guidelines including clinical examination and CT-scan or abdominal ultrasound every six months.

### 2.3. Statistical Analysis

The objective of the study was to compare survival of patients following pCT or aCT, according to histology in each group (pCT or a CT). The primary endpoint was OS and the secondary endpoints were EFS and tumor regression grade (TRG) for pCT. OS was defined as the time from the onset of treatment for pCT group or from surgery for aCT group to the date of death due to any cause, or censored at the date of last follow-up for alive patients. EFS was defined as the time from the start of treatment for pCT group or from surgery for aCT group to the date of the first documented recurrence or progression (local, regional or distant), death due to any cause or discontinuation of treatment for any reason, whichever occurred first. TRG was categorized according to the Mandard classification system [23]. The Kaplan–Meier method was used to estimate OS and EFS, a Cox regression model was employed to estimate hazard ratios (HRs) and two-sided 95% confidence intervals (CIs) were used for the comparison of survival of diffuse vs. intestinal subtype in both perioperative and adjuvant setting. Pearson chi-square test was performed for comparing TRG rate of intestinal and diffuse GCs, respectively, within pCT cohort. The statistical significance level was set at *p* < 0.05. Univariate analysis was performed to establish the relationship among survival endpoints and clinic-pathologic variables: age (<65 vs. >65 years), tumor location (proximal vs. distal), histology (diffuse vs. intestinal), lymphatic vascular infiltration (LVI) (absent vs. present), lymph node involvement (N0 vs. N+), grading (G1–G2 vs. G3), resection margin (R0 vs. R1), CT regimen employed (doublet vs. triplet) and exposure to adjuvant radiotherapy (RT) (absent vs. present). Clinical variables with a *p* value < 0.5 were included in a multivariate analysis. Data were analyzed using MedCal Statistical software.

## 3. Results

### 3.1. Patients Characteristics 

**Figure 1:**
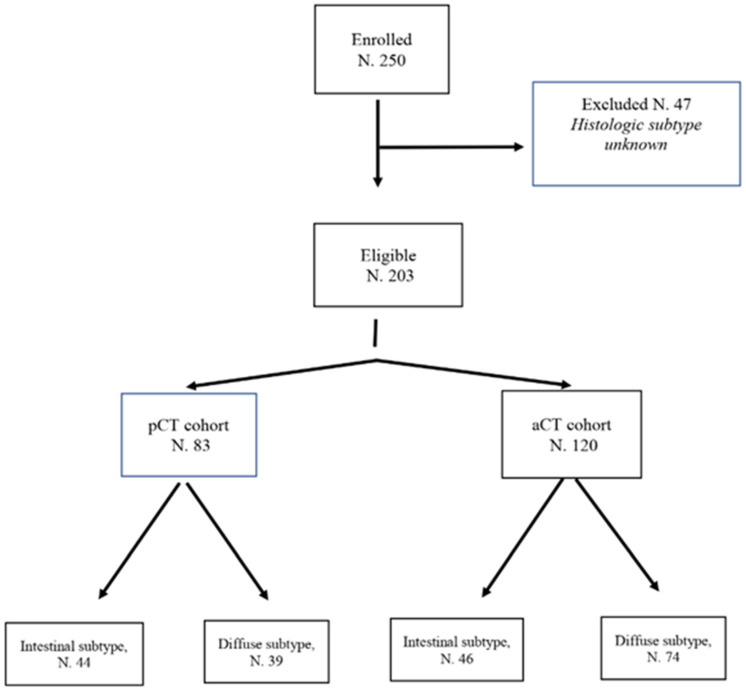
Consort diagram.

Among 250 consecutive patients affected by stage II or III GC treated at Medical Oncology Unit of the Fondazione Policlinico Universitario “A. Gemelli”–Comprehensive Cancer Center (IRCCS) between January 2009 to January 2018, 203 met all inclusion criteria and were included in this retrospective analysis (Figure 1). Forty-seven were excluded due to incomplete clinical and/or histologic information. Eighty-three patients underwent pCT and 120 received upfront surgery followed by aCT. One hundred thirteen patients were affected by diffuse GC, 39 were treated with pCT and 74 with surgery followed by aCT. Ninety patients had intestinal GC, 44 received pCT and 46 underwent upfront surgery followed by aCT. Within the whole population, 52% of patients (105) were male and 48% (98) were female. Median age was 64 years (range 42–78). A diagnostic laparoscopy was performed in 70% of cases to exclude peritoneal carcinomatosis in the pCT cohort. All patients (203) received an adequate D2 lymphadenectomy with a median of 38 lymph nodes evaluated (range 20–60). 

In the pCT group, patients diagnosed with a GEJ or stomach cancer were well-balanced between intestinal and diffuse sub-groups. In this group, four patients (4.8%) had a microscopic residual after surgery (R1); all of them belonged to the diffuse sub-group. The percentage of patients with a postoperative lymph node N0 status was higher (32.5% vs. 6.0%), when comparing intestinal and diffuse histology while the rate of patients with LVI was inferior (9.6% vs. 20.1%) in patients with an intestinal histology. Moreover, the cases of yT4 tumors were fewer (6.0% vs. 19.2%) among intestinal cancers in comparison to diffuse ones. In the pCT cohort, 24 patients received RT for R1 surgery (4.8%) or postoperative lymph node N3 status (24%), of whom 4 (4.8%) had an intestinal histology and 20 (24.0%) a diffuse cancer. 

In the pCT cohort, the preferred treatment regimens were in the intestinal subgroup EOX or ECF in 26.5% of patients and CF or FOLFOX in other 26.5%, whereas in the diffuse group 34.9% and 12.0%, respectively. Patients received a median of three cycles with EOX, ECF or CF (range 2–4) and a median of six cycles with FOLFOX (range 4–8). No dose reduction was required. Surgery was performed on average 38 days (range 28–54) after the last administration of chemotherapy. In the pCT cohort 36% of patients did not receive any treatment after surgery due to postoperative worsening of PS or poor response to preoperative CT. 

In the aCT group, patients diagnosed with a GEJ cancer were well-balanced between intestinal and diffuse histology. Among those with a stomach cancer, diffuse histotype was more frequent (27.5% vs. 49.1%). Even in this group, four patients (3.3%) had a microscopic residual after surgery (R1); all of them belonged to the diffuse sub-group once again. LVI was less frequent among patients with an intestinal cancer in comparison with those with a diffuse histology (19.1% vs. 30.8%). The cases of pT4 tumors were slightly more frequent in diffuse histology (30.0% vs. 17.5%) but there were no differences concerning patients with N0 status (4.8% vs. 2.4%). The percentage of patients receiving RT after surgery was higher in diffuse-type than intestinal histology (15% vs. 28.3%), due to a higher rate of R1 surgery and lymph node N3 status.

In the aCT cohort, cytotoxic therapy started on average 52 days (range 38–62) after surgery. In this case, the preferred regimens were: in the intestinal cohort, EOX/ECF (11.6%), FOLFOX or CF (12.5%) and DeGramont regimen (14.1%), while, in the diffuse cohort, 29.1%, 14.1% and 18.3%, respectively. Drug reductions were necessary in 25% of patients mainly due to neutropenia or diarrhea. The median follow-up time of whole group of patients was 41 months. Baseline patients and disease characteristics according to histology and treatment strategy are summarized in Table 1.

### 3.2. Outcome

**Figure 2:**
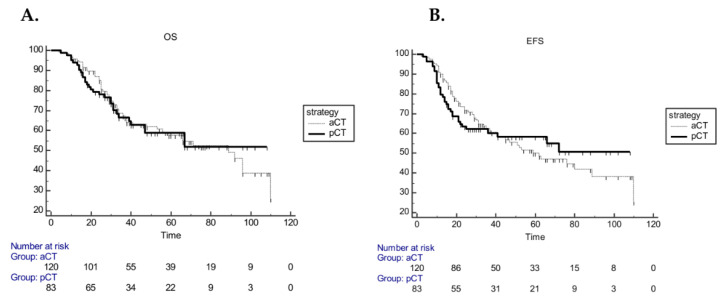
Kaplan–Meier curves for OS (**A**) and EFS (**B**) according to strategy (pCT vs. aCT).

Overall, median OS (mOS) and median EFS (mEFS) were 92 (range 66–110) and 66 (range 45–110) months, respectively. In the pCT cohort, mEFS and mOS were not reached, whereas, in the aCT cohort, mOS and mEFS were 89 (range 56–110 months) and 62 (range 41–110) months, respectively. No statistically significant difference was found both in mOS (*p* = 0.99) and EFS (*p* = 0.96) (Figure 2).

**Figure 3:**
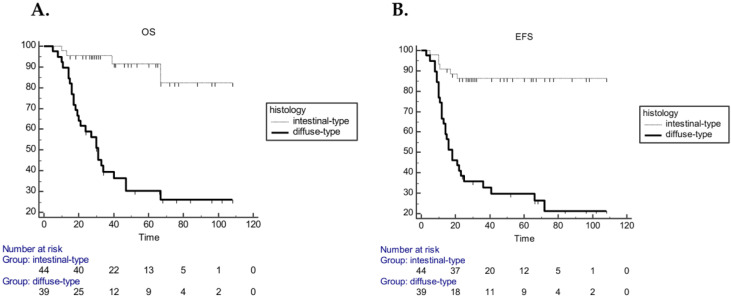
Kaplan–Meier curves for OS (**A**) and EFS (**B**) in the pCT cohort according histology (intestinal vs. diffuse subtype, HR 9.3; 95% CI 4.59–19.13; *p* < 0.0001 for OS; HR 7.2; 95% CI 3.6–14.27; *p* < 0.0001 for EFS).

In the pCT cohort, univariate Cox regression analysis, including age, tumor location, histology, LVI, lymph node involvement, grading, resection margin, CT regimen and exposure to adjuvant RT, demonstrated that grading, histology, LVI, resection margin and lymph node status were significantly associated with both EFS and OS (Table 2). Multivariate analysis performed on these variables demonstrated that only histology and LVI remained significantly associated with EFS (*p* = 0.0023; *p* = 0.0028) and OS (*p* = 0.0001; *p* = 0.0004) (Table 3). Median OS was 31 months (20–47) in the diffuse-type and not reached in the intestinal-type (HR 9.3; 95% CI 4.59–19.13; *p* < 0.0001), whereas the mEFS was 18 months (13–36) in the diffuse cohort and not reached in the intestinal-type (HR 7.2; 95% CI 3.6–14.27; *p* < 0.0001) (Figure 3).

**Figure 4:**
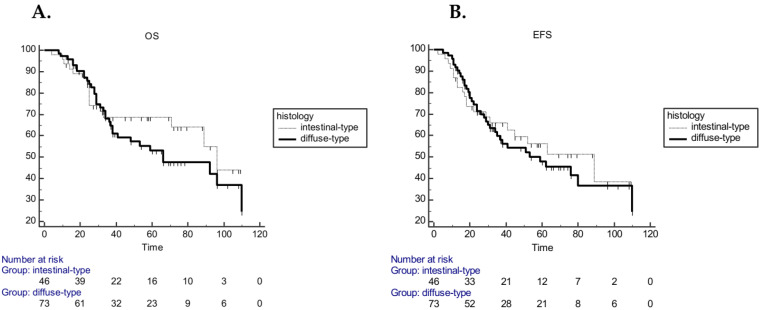
Kaplan–Meier curves for OS (**A**) and EFS (**B**) in aCT cohort according to histology (intestinal diffuse sub–type (HR 1.2; 95% CI 0.72–2.26; *p* = 0.40 for OS; HR 1.12 CI 95% 0.65–1.91; *p* = 0.67 for EFS).

In the aCT cohort, univariate analysis including the same variables showed that resection margin was significantly associated with both EFS and OS, whereas tumor location was associated only with EFS (Table 2). In multivariate analysis, resection margin confirmed the significant association with both EFS (*p* = 0.0082) and OS (*p* < 0.001) and also tumor location confirmed the relationship with EFS (*p* = 0.013) (Table 3). In the aCT cohort no statistically significant survival difference in relation to histology was observed. Median EFS was 89 months (range 41–89) in the intestinal subgroup and 59 months (range 35–110) in diffuse subtype (HR 1.12 CI 95% 0.65–1.91; *p* = 0.67), while mOS was 96 (range 71–96) and 66 (range 38–110) months, respectively (HR 1.2; 95% CI 0.72–2.26; *p* = 0.40) (Figure 4).

Moreover, in the perioperative cohort, a different percentage of tumor regression grade (TRG) was also observed according to histology, with a higher response in intestinal GC compared to diffuse GC. Over 60% of patients in the intestinal sub-group showed a TRG between 1 and 3 compared to diffuse-type, in which a TRG between 4 and 5 was obtained in over 60% of patients (*p* = 0.01). No differences were reported in TRG according different pCT regimens. 

## 4. Discussion

To our knowledge, this is the first report in real life suggesting the possibility of a histology-driven approach to the treatment of LAGC. Although retrospective and small-sized, this study generates the hypothesis that pCT could be the option of choice for patients with stage II–III intestinal gastric cancer, since they survive much longer when compared to patients with a diffuse histology receiving pCT. This finding does not seem due only to the known prognostic negative effect of diffuse histology, since, among patients treated in an adjuvant setting, there was no statistically significant difference concerning OS and EFS between diffuse and intestinal histology. Thus, the strategy of therapy (pCT vs. aCT) seems to have affected the outcome in relation to histotype. 

In the whole population of our study, there is no survival difference among patients in pCT cohort in comparison to those in aCT cohort. This is in contrast with some previous reports suggesting that five-year survival is lower with perioperative in comparison to adjuvant therapy [4,5,6,9,10]. However, the high quality of surgery with 100% of patients receiving D2 gastrectomy in the same institution together with accurate staging before surgery (70% laparoscopy rate) might account for this result, strengthening the acknowledged crucial role of surgery in this disease [24]. Interestingly, the pCT diffuse histology group showed the worst median OS (31 months) among subgroups, suggesting that stage migration during pCT or underestimation of stage of disease might have played a role for this disappointing outcome. 

Even if there is no definitive argument explaining our results, we might think that the alternative therapeutic strategies that have been considered might affect subtle molecular and biological differences between histologic subtypes [15]. Updated analysis of INT-0116 shows that intestinal subtype of gastric cancer takes advantage from aCT-RT, but not diffuse histotype, suggesting a different sensitivity to chemotherapy or an intrinsically worse prognosis [25]. More recently, the FLOT4 study has demonstrated the superiority of a triplet-regimen combination with fluorouracil plus leucovorin, oxaliplatin and docetaxel versus fluorouracil or capecitabine plus cisplatin and epirubicin in the perioperative setting of gastric cancer. In this study, which has become practice-changing superseding all previous trials of perioperative chemotherapy, patients have been stratified according histology [14]. Although the experimental approach appears equally effective in both histotype, HR for OS is much better in non-diffuse histology (0.74 vs. 0.85, *p* = 0.41). In contrast with our data, the JCOG 0501 phase III study has shown no statistically significant differences between pCT and aCT in poorly differentiated gastric cancer of an Eastern population, although this study and our study are difficult to be compared since the study has not been published in extenso yet and all patients received adjuvant chemotherapy [26].

The highest chemo-sensitivity of intestinal GC might allow a deeper response to neoadjuvant chemotherapy, which, in turn, could lead to smaller tumors at time of surgery, easier resection with a lower risk of R1 surgery, better control of micrometastatic disease and, ultimately, better outcomes. On the other hand, it might be feasible that in diffuse GC delayed surgery in favor of neoadjuvant chemotherapy, through unknown modifications of tumor microenvironment, could favor tumor cells extravasation and metastasis. Recently, another retrospective analysis suggested an upfront surgery approach highlighting no survival benefit from a pCT strategy in signet-ring cell carcinoma population [27]. 

Unfortunately, no randomized trial of pCT has evaluated response or survival in histologic subtypes, thus we cannot support our arguments with results of clinical research. However, it should be noticed that in our analysis the rate of patients without postoperative lymph nodes involvement was much higher in patients with intestinal subtype receiving pCT compared not only to patients with diffuse GC (32.5% vs. 6.0%) but also to patients with intestinal GC receiving aCT (32.5% vs. 3.3%). These observations, together with the higher rate of tumor regression observed in intestinal GCs compared to diffuse GCs, suggest that pCT is much more efficacious in patients with intestinal rather than diffuse GCs. In multivariate analysis, not only the histologic subtype, but also LVI resulted significantly associated with survival in the pCT cohort. Interestingly, the percentage of LVI was about 20–30% in all subgroups of both cohorts with the exception of intestinal subgroup in the pCT cohort (9.6%). Since there is no reason to believe that in such subgroup a population with a different biology had been casually selected, we think that it might result from a higher ability of pCT to reduce LVI (as well as lympho-nodes involvement) in the intestinal but not in the diffuse subtype. In addition, in all subgroups of our series, about 15–28% of patients received adjuvant RT, with the only exception of patients with intestinal GC treated with pCT. In this subgroup, only 4.8% of patients received adjuvant RT, mainly because the incidence of postoperative node-negative tumors was higher. Taken together, these observations suggest that a histology-driven approach may have an impact not only on the choice of CT strategy, but also modulate the therapeutic program, allowing to reduce the need of adjuvant RT in a subgroup of patients, thus avoiding useless toxicity and saving costs.

We are aware that these results have to be confirmed in larger analyses. We also know that in the present genomic era a histology-driven approach to crucial decisions looks simplistic and rather obsolete. However, the increasing cost of new drugs and technology might draw oncologists’ attention to this easy, inexpensive and widely available tool for improving the management of LAGC while waiting for more compelling indications coming from molecular research.

## 5. Conclusions

Our retrospective analysis seems to suggest that in a Western LAGC population the intestinal histotype might have a better efficacy from a pCT strategy compared to diffuse-type to whom an aCT approach might ensure better survival. These results have to be taken with wariness and no conclusive consideration are allowed.

## Figures and Tables

**Table 1:** Patients’ Characteristics.

Characteristic	Perioperative Chemotherapy (No. 83)			Characteristic	Adjuvant Chemotherapy (No. 120)		
	No.	Intestinal (No. 44)	Diffuse (No. 39)		No.	Intestinal (No. 46)	Diffuse (No. 74)
Tumor location				Tumor location			
GEJ	41	23 (27.7%)	18 (21.7%)	GEJ	28	13 (10.8%)	15 (12.5%)
Stomach	42	21 (25.3%)	21 (25.3%)	Stomach	92	33 (27.5%)	59 (49.2%)
Signet-cell	9	-	9 (10.8%)	Signet-cell	18	-	18 (15.0%)
LVI	25	8 (9.6%%)	17 (20.4%)	LVI	60	23 (19.1%)	37 (30.8%)
Total gastrectomy	41	19 (22.8%)	22 (26.5%)	Total gastrectomy	48	21 (17.5%)	27 (32.5%)
R1 surgery	4	-	4 (4.8%)	R1 surgery	4	-	4 (3.3%)
T				T			
yT0/T1	8	6 (7.2%)	2 (2.4%)	pT0/T1	4	1 (0.8%)	3 (2.5%)
yT2	16	12 (14.5%)	4 (4.8%)	pT2	13	6 (5.0%)	7 (5.9%)
yT3	38	21 (25.3%)	17 (20.5%)	pT3	46	18 (15.0%)	28 (23.3%)
yT4	21	5 (6.0%)	16 (19.3%)	pT4	57	21 (17.5%)	36 (30.0%)
N				N			
yN0	32	27 (32.5%)	5 (6.0%)	pN0	6	4 (3.3%)	2 (1.7%)
yN1	15	8 (9.7%)	7 (8.4%)	pN1	24	7 (5.8%)	17 (14.2%)
yN2	16	5 (6.0%)	11 (13.3%)	pN2	32	17 (14.2%)	15 (12.5%)
yN3	20	4 (4.8%)	16 (19.3%)	pN3	58	18 (15.0%)	40 (33.3%)
TRG							
TRG1	7	5 (6.0%)	2 (2.4%)				
TRG2	7	7 (8.4%)	-				
TRG3	28	17 (20.5%)	11 (13.3%)				
TRG4	34	15 (18.1%)	19 (22.9%)				
TRG5	7	-	7 (8.4%)				
Treatment				Treatment			
EOX/ECF	51	22 (26.5%)	29 (34.9%)	EOX/ECF	49	14 (11.6%)	35 (29.2%)
FOLFOX/CF	32	22 (26.5%)	10 (12.1%)	FOLFOX/CF	32	15 (12.5%)	17 (14.2%)
DeGramont	-	-	-	DeGramont	39	17 (14.2%)	22 (18.3%)
Radiotherapy	24	4 (4.8%)	20 (24.0%)	Radiotherapy	52	18 (15%)	34 (28.3%)

**Table 2:** Univariate Analysis of OS and EFS for Clinicopathologic Variables in pCT and aCT Cohort.

Variable	OS		EFS	
	HR (95% CI) for Mortality	*p* Value	HR (95% CI) for Progression	*p* Value
pCT cohort				
Age	0.6 (0.3–1.22)	0.1	0.4 (0.21–0.82)	0.01
Grading	2.7 (1.31–5.68)	0.004	3.2 (1.5–6.5)	0.0003
Histology	0.1 (0.05–0.21)	<0.0001	0.13 (0.07–0.27)	<0.0001
LVI	3.6 (1.55–8.34)	0.0001	3.8 (1.72–8.62)	<0.0001
Surgery R1	3.4 (0.71–16.9)	0.006	3.59 (0.71–18.0)	0.004
Tumor location	1.2 (0.61–2.53)	0.53	1.16 (0.59–2.26)	0.65
N status	4.71 (2.30–9.65)	0.0012	3.66 (1.87–7.15)	0.0017
Doublet vs. triplet chemotherapy	1.28 (0.64–2.54)	0.48	1.22 (0.58–2.58)	0.5
Radiotherapy	1.99 (0.91–4.1)	0.06	1.7 (0.86–3.68)	0.08
aCT cohort				
Age	1.70 (0.95–3.02)	0.06	1.48 (0.87–2.54)	0.12
Grading	1.31 (0.73–2.36)	0.37	1.45 (0.83–2.52)	0.19
Histology	1.28 (0.72–2.26)	0.40	1.12 (0.65–1.91)	0.67
LVI	1.31 (0.70–2.43)	0.66	1.13 (0.63–2.0)	0.66
Surgery R1	4.04 (0.58–27.9)	0.0033	13.2(0.61–28.01)	<0.0001
Tumor location	0.59 (0.29–1.21)	0.09	0.55 (0.28–1.07)	0.03
N status	1.5 (0.29–7.67)	0.68	2.03 (0.49–8.34)	0.46
Doublet vs. triplet chemotherapy	1.11 (0.60–2.03)	0.72	1.16 (0.66–2.05)	0.57
Radiotherapy	1.42 (0.82–2.47)	0.19	1.44 (0.85–2.42)	0.16

**Table 3:** Multivariate Cox Regression Analysis of EFS and OS for Clinicopathologic Variables Resulted Significant in Univariate Analysis; pCT and aCT Cohort.

Variable	OS		EFS	
	HR (95% CI) for Progression	*p* Value	HR (95% CI) for Mortality	*p* Value
pCT				
Histology	10.95 (3.31–36.24)	0.0001	4.84 (1.76–13.29)	0.0023
LVI	5.57 (2.18–14.26)	0.0004	3.60 (1.56–8.32)	0.0028
aCT				
Tumor location	–	–	0.47 (0.26–0.85)	0.013
Resection margin	19.97 (7.04–56.59)	<0.001	4.036 (1.44–11.30)	0.0082
